# Association between viral load and positivization time of a SARS-CoV-2 rapid antigen test in routine nasopharyngeal specimens

**DOI:** 10.5937/jomb0-35482

**Published:** 2022-10-15

**Authors:** Gian Luca Salvagno, Brandon M. Henry, Nitto Simone de, Laura Pighi, Giuseppe Lippi

**Affiliations:** 1 University of Verona, Section of Clinical Biochemistry, Verona, Italy; 2 Pederzoli Hospital, Service of Laboratory Medicine, Peschiera del Garda, Italy; 3 Cincinnati Children's Hospital Medical Center, Division of Nephrology and Hypertension, Clinical Laboratory, Cincinnati, United States of America; 4 Texas Biomedical Research Institute, Disease Intervention & Prevention and Population Health Programs, San Antonio, United States of America

**Keywords:** Covid-19, Sars-CoV-2, laboratory medicine, diagnosis, immunoassay, Covid-19, Sars-CoV-2, laboratorijska medicina, dijagnoza, imunoodre|ivanje

## Abstract

**Background:**

Rapid SARS-CoV-2 antigen tests are potentially useful tools for screening carriers with high viral load. This study was aimed to assess the potential association between viral load and positivization time of a manual SARS-CoV-2 commercial antigen test in routine nasopharyngeal specimens.

**Methods:**

In a sample of subjects undergoing routine diagnostic testing, SARS-CoV-2 positivity of nasopharyngeal samples was assayed with both molecular (Altona Diagnostics RealStar SARS-CoV-2 RT-PCR Kit) and antigenic (Roche SARS-CoV-2 Rapid Antigen Test) tests. Positivization time of rapid antigen test was correlated and compared with viral load expressed as mean of SARS-CoV2 E/S genes cycle threshold (Ct) values.

**Results:**

The study sample consisted of 106 patients (median age 48 years, 55 women) with positive results of rapid SARS-CoV-2 antigen testing. A highly significant Spearman's correlation was found between mean SARSCoV-2 E/S genes Ct values and positivization time of manual antigen test (r= 0.70; p<0.001). The positivization time of rapid SARS-CoV-2 antigen test displayed an area under the curve of 0.82 (95%CI, 0.74-0.89) for predicting nasopharyngeal samples with high viral load (i.e., mean Ct <20). A positivization time cut-off of 32 SEC had 94.9% sensitivity and 58.2% specificity for detecting specimens with high viral load. The overall agreement between mean Ct value <20 and positivization time <32 SEC was 70.8%.

**Conclusions:**

Positivization time of rapid SARS-CoV-2 antigen tests may provide easy and rapid information on viral load, thus making this type of manual assay potentially suitable for quick and reliable detection and isolation of supercarriers.

## Abstract (sr)

**Uvod:**

Brzi testovi na SARS-CoV-2 antigen su potencijalno korisni alati za skrining nosilaca sa visokim virusnim opterećenjem. Ova studija je imala za cilj da proceni potencijalnu povezanost između virusnog opterećenja i vremena pozitivanosti manuelnog SARS-CoV-2 komercijalnog testa antigena u rutinskim uzorcima nazofarinksa.

**Metode:**

U uzorku subjekata koji su bili podvrgnuti rutinskom dijagnostičkom testiranju, SARS-CoV-2 pozitivnost nazofaringealnih uzoraka je analizirana i molekularnim (Altona Diagnostics RealStar SARS-CoV-2 RT-PCR komplet) i antigenskim (Roche SARS-CoV-2 Rapid Antigen Test) testovima. Vreme genskog brzog testa na antigen je u korelaciji i upoređeno sa virusnim opterećenjem izrađenim kao srednja vrednost praga ciklusa (Ct) SARS-CoV-2 E/S.

**Rezultati:**

Uzorak studije sastojao se od 106 pacijenata (srednje starosti 48 godina, 55 žena) sa pozitivnim rezultatima brzog testiranja na SARS-CoV-2 antigen. Pronađena je visoko značajna Spirmanova korelacija izmenu srednjih vrednosti Ct gena SARS-CoV-2 E/S i vremena pozitivnosti ručnog testa na antigen (r= 0,70; p<0,001). Vreme pozitivnosti brzog testa SARS-CoV-2 antigena pokazalo je područje ispod krive od 0,82 (95% CI, 0,74-0,89) za predviđanje nazofaringealnih uzoraka sa visokim virusnim opterećenjem (tj. srednji Ct <20). Prekid vremena pozitivnosti od 32 sekunde imao je 94,9% osetljivosti i 58,2% specifičnosti za detekciju uzoraka sa visokim virusnim opterećenjem. Ukupna saglasnost između srednje vrednosti Ct <20 i vremena pozitivnosti <32 SEC bila je 70,8%.

**Zaključak:**

Vreme pozitivnosti brzih testova na antigen SARSCoV-2 može pržiti lake i brze informacije o virusnom opterećenju, čineći tako ovaj tip manuelnog testa potencijalno pogodnim za brzo i pouzdano otkrivanje i izolaciju super-nosača.

## Introduction

Although molecular identification of severe acute respiratory syndrome coronavirus 2 (SARS-CoV-2) RNA remains the reference technique for diagnosing coronavirus disease 2019 (COVID-19) infection, the use of laboratory-based immunoassays or manual (rapid) antigen tests is rapidly growing around the world [1]. Rapid immunoassays for detecting SARS-CoV-2 antigens (especially the nucleocapsid protein; N) have some obvious advantages, but also some inherent limitations. Major rapidity and relatively lower costs compared to laboratory-based molecular detection, along with possibility to (self-) usage outside the laboratory environment are the most apparent benefits of manual SARS-CoV-2 antigen tests, whilst visual interpretation of test results, generation of qualitative data (i.e., positive/negative) and limited accuracy (i.e., especially suboptimal analytical and diagnostic sensitivity), are well-known drawbacks of these tests [2] [3] [4].

Due to the immense number of diagnostics tests that are still needed within the timeframe of the ongoing COVID-19 pandemic, and due to a reliable prevision that such volume may further increase with progressive diffusion of highly mutated and more infectious lineages such as the new Omicron (B.1.1.529) variant [5], rapid antigen tests can be considered a potentially useful screening tool, especially suited for identification of individuals bearing high nasopharyngeal SARS-CoV-2 viral loads. These patients, who are conventionally called »super-carriers« and »super-spreaders«, may be responsible for a disproportionate number of secondary infections compared to an »average« infectious individual, due to the higher volume of viral particles that they could disseminate in the environment [6]. A high number and heterogeneity of interpersonal contacts, along with high viral load in the upper and/or lower respiratory tracts, are currently considered the main drivers of super-spreading events [7], such that is has been estimated that an accurate and timely identification of these super-carriers would allow prevention of up to 90% of all secondary SARS-CoV-2 infections [8].

Given that the diagnostic accuracy of rapid SARS-CoV-2 antigen tests depends on the viral load present in the test samples, displaying diagnostic sensitivity as high as 95% in those with cycle threshold (Ct) values 25 [9] [10] and sensitivity up to 99% versus positive viral cultures [11], this study was designed to assess whether the positivization time of a manual SARS-CoV-2 antigen commercial immunoassay could provide useful information on viral load in routinely collected nasopharyngeal specimens.

## Materials and methods

The study population consisted of a series of patients presenting to the Service of Laboratory Medicine of Hospital Pederzoli (Peschiera del Garda, Verona, Italy) for COVID-19 testing (either for being symptomatic or in close contact with SARS-CoV-2 positive subjects), between December 1 and 20, 2021. A routine nasopharyngeal swab (Virus swab UTM Copan, Brescia, Italy) was collected upon admission and immediately transported to the Laboratory Medicine Service, for being assayed with both antigen and molecular testing, in compliance with locally defined standard operating procedures (SOPs).

Molecular testing for SARS-CoV-2 RNA detection was carried out with the Altona Diagnostics RealStar SARS-CoV-2 RT-PCR Kit (Altona Diagnostics GmbH, Hamburg, Germany), a real-time reverse transcription polymerase chain reaction (rRT-PCR) technique encompassing double concomitant amplification of SARS-CoV-2 *S* and *E* genes. All tests were performed using Bio-Rad CFX96™ Deep Well Dx Real-Time PCR Detection System (Bio-Rad Laboratories, Hercules, CA, USA). The limit of detection of this technique is 3.8 SARS-COV-2 copies/mL [12]. Test results are considered analytically positive when the Ct values of both SARS-CoV-2 *E* and *S* genes are both <45, whilst infectivity is associated with Ct values of both genes <29.5 (i.e., threshold of viral culture positivity) [13].

Rapid SARS-CoV-2 antigen testing was performed using the Roche SARS-CoV-2 Rapid Antigen Test (Roche Diagnostics SpA, Monza, Italy), whose technical characteristics and analytical performance have been previously described elsewhere [14]. Briefly, nasopharyngeal samples are collected and deposited within an extraction buffer container. Three drops of material are then applied to the reagent tray. When viral antigens are present in sufficient concentration in the specimen, they are bound to specific monoclonal anti-SARS-CoV-2 mouse antibodies, and such process is mirrored by appearance of a coloured line in the result window, together with concomitant appearance of another »control« band in the upper part of the device. The Limit of Detection is 3.12×102.2 TCID _50_/mL, whilst diagnostic sensitivity and specificity were found to be as high as 73% and 99%, respectively [14]. According to manufacturer's recommendations, test results shall be read between 15-30 min.

Results of molecular testing were expressed as median Ct values (and interquartile range; IQR), whilst those of the rapid antigen test were classified as positive upon appearance of two clear bands in both the reactive and control windows of the device. The time from positivization was measured using a standard digital chronometer, as the time passed between addition of the last drop of test reagent and appearance of the positive band in the reactive window.

The association between positivization time of rapid antigen test and viral load expressed as mean of SARS-CoV-2 E/S genes Ct values ((SARS-CoV-2 *E* Ct value + SARS-CoV-2 *S* Ct value)/2) was analyzed with Spearman's correlation and compared with Kappa test for inter-rater agreement and receiving operating characteristics (ROC) curve. The statistical analysis was carried out with Analyse-it software (Analyse-it Software Ltd, Leeds, UK). This study was part of routine clinical laboratory operations for SARS-CoV-2 screening and diagnosis at the local facility, such that patient informed consent and Ethical Committee approval were unnecessary. The study was conducted in accordance with the Declaration of Helsinki, under the terms of relevant local legislation.

## Results

**Figure 1:**
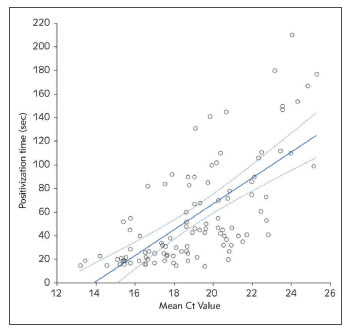
Spearman’s correlation between positivization time of a manual SARS-CoV-2 antigen test and viral load expressed as mean cycle threshold (Ct) value of SARS-CoV-2 E and S genes in routine nasopharyngeal specimens.

The final study population consisted of 106 patients (median age 48 years, IQR 30-68 years; 55 women) following the exclusion of 615 patients with negative results on the rapid antigen test, whose samples were hence unusable for evaluating the strength of association between viral load and positivization time. The median Ct values in the study population samples were 19.2 (IQR, 17.3-21.2) and 18.8 (IQR, 16.6-20.4) for the SARS-CoV-2 *E* and *S* genes, respectively (the median of the mean value of both genes in each specimen was 19.0; IQR 17.0-20.7). A total number of 39/106 samples (36.8%) displayed especially high viral load (i.e., mean Ct of both SARS-CoV-2 *E* and *S* genes <20). The median time of positivization of the manual antigen test was 42 (IQR, 23-81; range, 14-210) sec. A highly significant Spearman's correlation was found between the mean SARS-CoV-2 *E/S* genes Ct values and positivization time of manual antigen test (r= 0.70; 95%CI, 0.59-0.79; p<0.001) (Figure 1).

**Figure 2:**
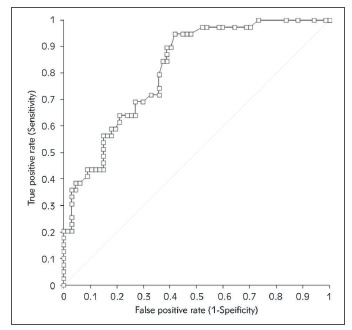
Receiving Operating Characteristics (ROC) curve analysis of the positivization time of a manual SARS-CoV-2 antigen test to predict mean cycle threshold (Ct) values ≤20 of SARS-CoV-2 E and S genes in routine nasopharyngeal specimens.

The accuracy of positivization time of the rapid SARS-CoV-2 antigen test for predicting nasopharyngeal samples with high viral load (i.e., mean Ct <20) is summarized in Figure 2, showing considerably high area under the curve (AUC, 0.82; 95%CI, 0.74-0.89). The positivization time cut-off with the best performance for detecting nasopharyngeal specimens with high viral load (i.e., mean Ct <20) was 32 sec, displaying 94.9% sensitivity and 58.2% specificity. The overall agreement between mean Ct value <20 and positivization time <32 sec was 70.8% (kappa statistics, 0.44; 95%CI, 0.29-0.59).

## Discussion

The relentless emergence and spread of new SARS-CoV-2 variants characterized by enhanced virulence and infectivity are creating paramount challenges to all clinical laboratories worldwide [15], increasingly struggling to support public preventive measures based on rapid testing and accurate contract tracing [16]. Although recent evidence has suggested that strategies based on identification of the so-called super-carriers and/or super-spreaders may be advantageous or even essential for effective control of local SARS-CoV-2 outbreaks [13] [17], population screening could only be feasible using decentralized and rapid testing, which enables to process a high volume of specimens collected from people of all ages, outside conventional diagnostic facilities (i.e., at mass gatherings, public transportation, schools, airports and so forth) [18] [19].

Consolidated evidence now attest that the reliability and accuracy of rapid SARS-CoV-2 antigen tests are seemingly high in respiratory tract specimens collected from people with a high viral load. One of the traditional drawbacks of these manual immunoassays is that the viral load cannot be accurately defined, since only qualitative results can be generated (i.e., positive/negative). Nonetheless, the possible usage of these devices for obtaining semi-quantitative data may engender important clinical and social benefits, by enabling timely identification of positive samples characterized by high viral load, and thus allowing for rapid detection and isolation of super-carriers and (potential) super-spreaders [20].

The results of our investigation evidenced that the viral load in routinely collected nasopharyngeal samples is highly correlated with the positivization time of a rapid SARS-CoV-2 antigen test, displaying AUC as high as 0.82 and overall agreement at high viral load threshold (i.e., Ct <20) of nearly 71%. A similar study has been previously conducted by Akashi and colleagues, using the QuickNavi™ - COVID19 Ag (Denka Co., Ltd., Tokyo, Japan) [21]. Briefly, the authors found that the time to obtain positive results was also positively correlated with Ct values (p<0.001), such that specimens with higher Ct values of SARS-CoV-2 *N2* gene were significantly associated with a longer time to obtain a positive reaction and vice versa, ultimately displaying up to 91% concordance for identifying SARS-CoV-2 RNA positive specimens.

In conclusion, the findings of this study confirm that the use of a very simple and rapid strategy, such as measuring the positivization time of a rapid SARS-CoV-2 antigen test, may enable to garner potentially useful information on viral load of tested subjects, thus making this type of manual assays potentially suitable for quick and reliable detection and isolation of super-carries before participation in mass gatherings or other social events where the likelihood of virus spread is high.

## Dodatak

### Research funding

None declared.

### Author contributions

All authors have accepted responsibility for the entire content of this manuscript and approved its submission.

### Informed consent

Informed consent was obtained from all individuals included in this study.

### Acknowledgments

The authors acknowledge the staff of the Service of Laboratory Medicine of the Pederzoli Hospital (Peschiera del Garda, Italy) for the skill technical assistance.

### Conflict of interest statement

All the authors declare that they have no conflict of interest in this work.
